# Exploring racial-specific associations between the food inflammation index(FII) and metabolic dysfunction-associated fatty liver disease (MAFLD) Prevalence: Data from the National Health and Nutrition Examination Survey 1999–2020

**DOI:** 10.1097/MD.0000000000049152

**Published:** 2026-06-05

**Authors:** Shiyan Wang, Yu Wang, Hui Peng, Jin Wang, Enqiang Chen

**Affiliations:** a Department of Pharmacy, West China Hospital, Sichuan University, Chengdu, China; b Center of Infectious Diseases, West China Hospital, Sichuan University, Chengdu, China.

**Keywords:** cross-sectional study, food inflammation index, metabolic dysfunction-associated fatty liver disease, NHANES

## Abstract

The Food Inflammation Index (FII) is a novel indicator for assessing the impact of diet on systemic inflammation. Unlike the Dietary Inflammation Index, which focuses on nutrients, the FII quantifies specific foods, providing greater applicability to real-world dietary assessments. To date, no study has examined the association between FII and the prevalence of Metabolic dysfunction-associated fatty liver disease (MAFLD). This study aimed to investigate the relationship between FII and MAFLD. This cross-sectional study included 25,067 individuals aged ≥ 20 years from the 1999 to 2020 National Health and Nutrition Examination Survey (NHANES). Univariate and multivariate logistic regression analyses were performed to explore the relationship between FII and MAFLD. Nonlinear associations between FII and MAFLD were examined using restricted cubic spline (RCS) analysis. Subgroup analyses, interaction tests, and threshold effect analyses were conducted to assess differences across groups. The prevalence of MAFLD was 44.45%. FII was positively associated with MAFLD in all models (Models 1, 2, and 3, *P* < .05). In model 3, individuals in the highest tertile of FII had an odds ratio (OR) of 1.21 [95% confidence interval (CI): 1.11–1.32] for MAFLD, compared to those in the lowest tertile. Subgroup analyses and interaction tests revealed sex- and race-specific associations between FII and MAFLD. Among non-Hispanic whites, the optimal FII threshold for preventing MAFLD was found to be -11.56. A lower FII was associated with a lower prevalence of MAFLD. The impact of FII on MAFLD prevalence was more pronounced among non-Hispanic whites compared to other racial groups. An FII score of < -11.56 for daily food intake was associated with a reduced risk of MAFLD in non-Hispanic whites.

## 1. Introduction

Metabolic dysfunction-associated fatty liver disease (MAFLD) has emerged as a critical medical concept, redefining the traditional understanding of fatty liver disease by emphasizing its strong association with systemic metabolic dysregulation.^[1]^ Unlike the previous diagnostic framework of nonalcoholic fatty liver disease (NAFLD), MAFLD explicitly links hepatic steatosis: confirmed via histology, imaging, or blood biomarkers—to underlying metabolic dysfunction, requiring at least 1 of 3 metabolic abnormalities for diagnosis.^[2–4]^ This shift in nomenclature and diagnostic criteria underscored the central role of metabolic disturbances in disease pathogenesis and progression, reflecting a more precise and clinically relevant classification. With a global prevalence exceeding 30%, MAFLD has become a leading contributor to cirrhosis and hepatocellular carcinoma, particularly in developed nations.^[5,6]^

A growing body of evidence implicated chronic metabolic inflammation as a critical mediator in MAFLD development and progression.^[7,8]^ Pro-inflammatory cytokines (e.g., TNF-α, IL-6) and adipokines secreted by dysfunctional adipose tissue exacerbate hepatic insulin resistance, promote lipotoxicity, and trigger fibrogenic pathways.^[9–11]^ Importantly, this inflammatory milieu is modifiable, with dietary composition playing a key role in either amplifying or mitigating systemic inflammation.^[12,13]^ The dietary inflammatory index (DII) is a validated, literature-derived tool that quantifies the inflammatory potential of an individual diet based on its association with circulating inflammatory biomarkers.^[14]^ Pro-inflammatory diets (high in refined carbohydrates, saturated fats, and processed meats) are associated with elevated DII scores and have been linked to obesity, type 2 diabetes (T2DM), and cardiovascular diseases—conditions frequently comorbid with MAFLD.^[15,16]^ Numerous previous studies have shown a significant association between dietary inflammatory index and NAFLD or MAFLD.^[17–19]^ However, there are limitations to relying solely on DII without considering wider food intake. It is crucial to recognize the critical role of food intake in relation to inflammation in everyday life.^[20]^

To address this limitation, Li et al developed the Food Inflammation Index (FII), an algorithmic model that directly correlates food consumption patterns with inflammatory biomarker levels, offering a more intuitive assessment of dietary inflammatory potential.^[21]^

Given that the role of race in modulating the association between the FII and MAFLD remains underexplored, this study aims to reassess the association between FII and MAFLD from a racial perspective among the NHANES (National Health and Nutrition Examination Survey) population.

## 2. Materials and methods

### 2.1. Study population

**Figure 1.**
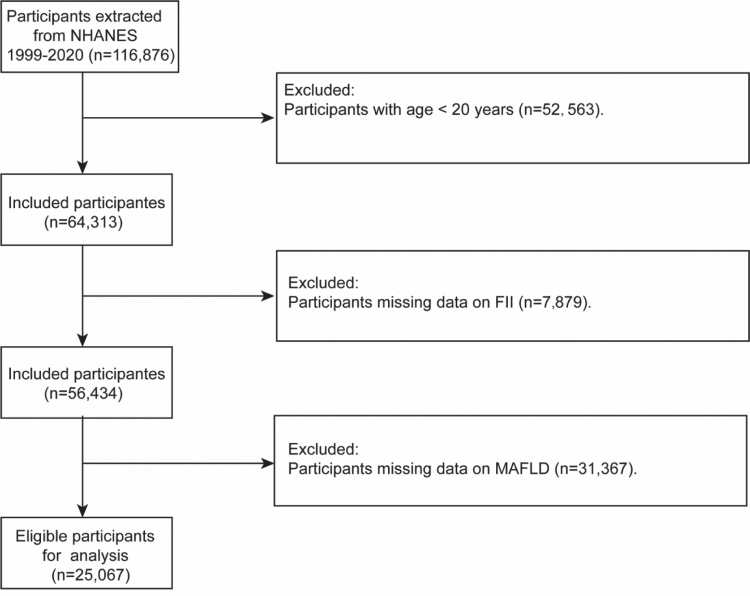
Diagram showing how the study population is selected.

Data for this study were obtained from the NHANES database, an annual survey conducted by the Centers for Disease Control and Prevention in the US, which uses a national, multistage, stratified cluster design to represent the noninstitutionalized civilian population. Participants provided written informed consent to the Ethics Review Board of the National Center for Health Statistics (NCHS). We selected data from NHANES (1999–2020), which recruited 116,876 participants. In our study, we excluded participants who younger than 20 years old (N = 52,563), those without FII data (N = 7879), and individuals without MAFLD data (N = 31,367) (Fig. 1). Finally, 25,067 eligible participants were included in the study.

### 2.2. Exposure variable

The identification and algorithm of FII are fully elaborated in the study of Li et al.^[21]^ The calculation of FII is based on the Food and Nutrition Diet Research Database (FNDDS) of the United States Department of Agriculture (USDA). Dietary intake data, including the types and quantities of foods and beverages consumed within the 24 hours preceding the interview, were recorded using a 24-hour dietary recall method. These data were collected through interviews conducted at the Mobile Examination Centers (MEC) as part of the NHANES. The collected data were used to calculate the FII, which is an adaptation of the DII and has been validated in large cohorts such as NHANES.

The calculation of FII is based on nutrients in 39 kinds of foods. These components include macronutrients, vitamins, minerals and fatty acids, such as omega-3 polyunsaturated fatty acids, fiber, as well as vitamins A, C and E. Each nutrient was assigned a Total Inflammation Score (TIS) based on published evidence of its pro- or antiinflammatory effects, along with a Nutrient Reference Value (NRV) obtained from the Dietary Guidelines for Americans (for individuals aged 31–50 years).^[15]^ The data in the Chinese Food Composition Table (China-FCT) is based on the “Chinese Food Composition Table Standard Edition, Sixth Edition” compiled by the Nutrition and Health Department of the Chinese Center for Disease Control and Prevention.^[22]^ The determination of FII is announced as follows:FII/100g = $\overset{n}{\underset{𝔦=1}{\sum }}\frac{TIS_{i}\times N_{i}}{NRV_{i}}$. Among them, FII is the food Inflammation Index; TIS_*i*_ is the total inflammatory score of each food component in FII; N_i_ is the amount of dietary components in every 100g of food; NRV_i_ is the recommended nutritional value in the Dietary Guidelines of the United States, with the corresponding value for the age range of 31 to 50 as the standard. To minimize the impact of outliers to the greatest extent, the scoring range of 9229 kinds of food was truncated at the 1% and 99% percentiles. The final FII score is scaled proportionally to the score range of all foods according to the following form ula, from 1 (the most pro-inflammatory) to 100 (the most antiinflammatory).The calculation formula is $FII\;\;\;score=100\text{-}abs\;\;\;\left(\frac{FII-FII\left(99\%\right)}{FII\left(1\%\right)-FII\left(99\%\right)}\right)\times 99.$ Among them, the FII score is the Food Inflammation Index score, FII is the food inflammation index, FII(99%) is the value of FII at the 99% percentile, and FII(1%) is the value of FII at the 1% percentile.

Furthermore, the FII is validated through its correlation with inflammatory biomarkers such as high-sensitivity C-reactive protein (hsCRP), confirming its predictive accuracy in assessing inflammation levels associated with dietary intake. Spearman correlation analysis is used to compare the FII with other dietary indices, such as the DII, to ensure its reliability.

### 2.3. Outcome variable

MAFLD was diagnosed per the 2020 National Expert Group Consensus, requiring both hepatic steatosis and at least 1 metabolic abnormality: overweight/obesity, T2DM, or metabolic dysregulation.^[3]^ Hepatic steatosis was identified through standardized ultrasonographic assessment, with severity classified into 4 grades. For analytical purposes, mild, moderate and severe cases were grouped as positive findings. The diagnostic protocol incorporated dual ultrasonography datasets from NHANES III to enhance reliability.^[23]^ This diagnostic approach emphasizes metabolic dysfunction while maintaining consistency with international standards for fatty liver disease identification. The combination of imaging evidence and metabolic criteria provides a clinically relevant framework for MAFLD.

### 2.4. Covariates

Potential confounders of the relationship between FII and MAFLD were taken into consideration for this study, including demographic factors, laboratory data, and data derived from the survey questionnaire. Analyzed participant demographics including age, sex, race, education, marital status, body mass index (BMI) and family income/poverty ratio (FIR).

Analyzed lifestyle factors included smoking status and excessive drinking. Smoking status was defined as smoking more than 100 cigarettes in adulthood.^[24]^ Excessive drinking was defined as ≥ 30 g/d for men or ≥ 20 g/d for women.^[25]^

Comorbid conditions that could impact MAFLD, such as cardiovascular disease (CVD), hypertension, and diabetes mellitus (DM), were also considered. Individuals with an SBP ≥ 130 mm Hg and/or DBP ≥ 80 mm Hg was defined as having hypertension. Meanwhile, participants who answered “yes” to the question: “Ever told you had high blood pressure?” are also classified as having hypertension.^[26]^ DM was defined according to: Physician-diagnosed diabetes; HbA1c > 6.5%; Fasting blood glucose ≥ 7.0 mmoL/L; Random blood glucose ≥ 11.1 mmoL/L; 2-hour Oral Glucose Tolerance Test (OGTT) blood glucose ≥ 11.1 mmoL/L; Use of diabetes medication or insulin.^[27]^ The diagnosis of CVD was established by self-reported physician diagnoses obtained during an individual interview using a standardized medical condition questionnaire. The participants were asked, “Has a doctor or other health expert ever informed you that you have congestive heart failure/coronary heart disease/angina pectoris/myocardial infarction/stroke?.” A person was regarded as having CVD if he or she replied “yes” to any of the above questions.^[28]^

### 2.5. Statistical analysis

Data were analyzed using R statistical software (version 4.1.3). The analysis included measures such as mean (standard deviation) for continuous variables, and frequencies and percentages for categorical variables. Group comparisons for continuous variables were performed using Welch t-test or ANOVA. For comparison between groups of categorical data, we used the Fisher exact test for expected frequencies < 5; otherwise, we used the Chi-squared test. To examine the association between FII and MAFLD, we performed univariate and multivariate logistic regression analysis. Three models were developed: Model 1 (unadjusted); Model 2 (adjusted for age, sex and race); and Model 3 (adjusted for age, sex, race, marital status, FIR, education, CVD, excessive drinking and smoking status). To further investigate this association, subgroup analysis and interaction analysis were performed. The dose-effect relationship and threshold effect analysis were used to assess group differences in this association.

## 3. Results

### 3.1. Basic characteristics of the population

Table 1 exhibited the basic characteristics of the 25,067 participants included in the study. The median age was 50 ± 18 years old, with 48.5% of participants being males and 51.5% females. Our analysis found that participants with MAFLD were more likely to be male, Mexican American, married, less educated, and poor. In addition, those participants were more likely to be obese, smoking, and have a higher prevalence of DM, hypertension, and CVD. Of note, FII was significantly different between participants with and without MAFLD.

**Table 1:** Patient demographics and baseline characteristics.

Characteristic	MAFLD			*P*-value
	Overall, N = 25,067*	No, N = 13,925*	Yes, N = 11,142*	
**Age (years**)	50 ± 18	48 ± 19	52 ± 16	<.001†
**Sex (%**)				<.001‡
Female	12,912 (51.5%)	7667 (55.1%)	5245 (47.1%)	
Male	12,155 (48.5%)	6258 (44.9%)	5897 (52.9%)	
**Race (%**)				<.001‡
Mexican American	4349 (17.3%)	2099 (15.1%)	2250 (20.2%)	
Non-Hispanic Black	5115 (20.4%)	2776 (19.9%)	2339 (21.0%)	
Non-Hispanic White	11,034 (44.0%)	6158 (44.2%)	4876 (43.8%)	
Other Hispanic	2181 (8.7%)	1192 (8.6%)	989 (8.9%)	
Other Race - Including Multi-Racial	2388 (9.5%)	1700 (12.2%)	688 (6.2%)	
**Marital status (%**)				<.001‡
Married/Living with Partner	13,214 (52.7%)	7204 (51.7%)	6010 (53.9%)	
Never married	7293 (29.1%)	4304 (30.9%)	2989 (26.8%)	
Widowed/Divorced/Separated	4560 (18.2%)	2417 (17.4%)	2143 (19.2%)	
**Education (%**)				<.001‡
˂ High school	6396 (25.5%)	3257 (23.4%)	3139 (28.2%)	
˃ High school	12,867 (51.3%)	7583 (54.5%)	5284 (47.4%)	
High school	5804 (23.2%)	3085 (22.2%)	2719 (24.4%)	
**FIR (%**)				
˂1.3	7446 (29.7%)	3921 (28.2%)	3525 (31.6%)	<.001‡
1.3–3.5	9785 (39.0%)	5375 (38.6%)	4410 (39.6%)	
˃3.5	7836 (31.3%)	4629 (33.2%)	3207 (28.8%)	
**BMI (kg/m^2^**)	29 ± 7	25 ± 3	34 ± 6	<.001†
**Excessive drinking (%**)				<.001‡
No	13,049 (52.1%)	7499 (53.9%)	5550 (49.8%)	
Yes	12,018 (47.9%)	6426 (46.1%)	5592 (50.2%)	
**Smoke status (%**)				<.001‡
No	13,553 (54.1%)	7932 (57.0%)	5621 (50.4%)	
Yes	11,514 (45.9%)	5993 (43.0%)	5521 (49.6%)	
**DM (%**)				<.001‡
No	20,251 (80.8%)	12,443 (89.4%)	7808 (70.1%)	
Yes	4816 (19.2%)	1482 (10.6%)	3334 (29.9%)	
**Hypertension (%**)				<.001‡
No	14,501 (57.8%)	9438 (67.8%)	5063 (45.4%)	
Yes	10,566 (42.2%)	4487 (32.2%)	6079 (54.6%)	
**CVD (%**)				<.001‡
No	22,318 (89.0%)	12,752 (91.6%)	9566 (85.9%)	
yes	2749 (11.0%)	1173 (8.4%)	1576 (14.1%)	
**FII**	−7.2 ± 6.5	−7.4 ± 7.1	−7.0 ± 5.8	<.001†

BMI = body mass index, CVD = cardiovascular disease, DM = diabetes mellitus, FII = food inflammation index, FIR = family income to poverty ratio, MAFLD = metabolic dysfunction-associated fatty liver disease.

* Mean ± SD; n (%).

† Welch 2 Sample t-test.

‡ Pearson’s Chi-squared test.

### 3.2. Logistic regression analysis of FII and MAFLD

**Figure 2.**
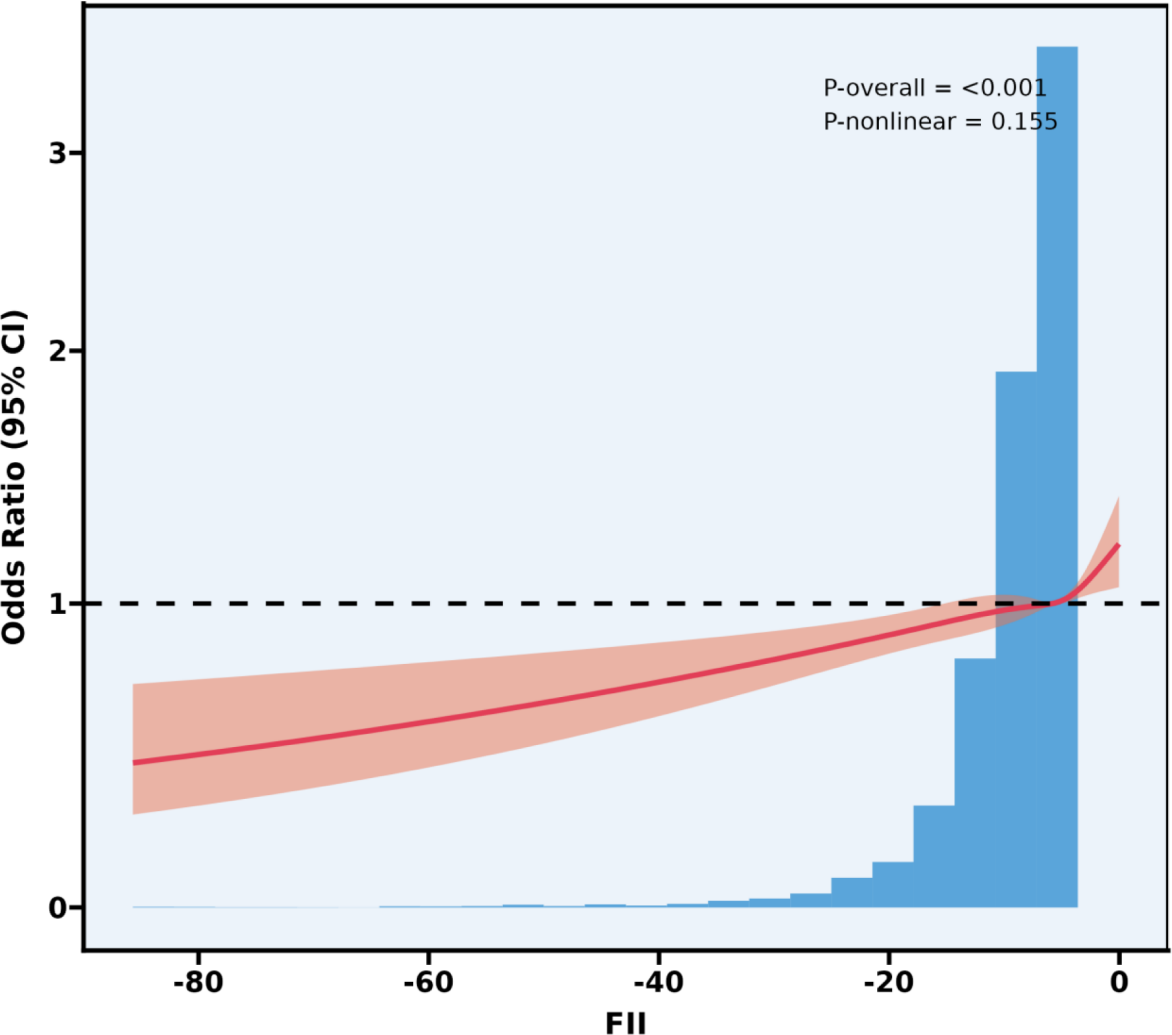
Associations between FII and MAFLD Prevalence: RCS Analysis.

We used multiple logistic regression models to validate the relationship between FII and MAFLD, and the results are shown in Table 2. In the unadjusted crude model (**Model 1**), FII was significantly positively associated with the risk of MAFLD when treated as a continuous variable (OR = 1.01, 95%CI 1.01–1.01, *P *< .001). After full adjustment for confounding factors, the correlation between FII and MAFLD risk was still positive (OR = 1.01, 95%CI 1.01–1.02, *P *< .001), which means that the association between FII and MAFLD was stable (**Model 3**). When FII was categorized, the medium and high tertiles demonstrated progressively higher risks of MAFLD compared to the lowest group. Specifically, the medium tertile showed ORs was 1.08 (95% CI 1.01–1.20, *P* = .015), while the high tertile exhibited ORs was 1.13 (95% CI 1.06–1.32, *P* < .001) across models. A significant dose-response trend was observed (*P* for trend < .001), indicating increasing MAFLD risk with higher FII levels. Adjustments for covariates, including age, sex, race, Marital status, FIR, education, CVD, excessive drinking, and smoke status, did not substantially alter these associations. These findings suggest that FII, particularly at higher levels, is a robust predictor of MAFLD risk. To further explore the relationship between FII and MAFLD, we performed a RCS analysis, with results shown in Figure 2. No nonlinear association was found between FII and MAFLD (*P*-value < .001, *P*-nonlinear = .107), which further supports the presence of an inverse linear relationship.

**Table 2:** Association between FII and MAFLD.

Characteristic	Model 1			Model 2			Model 3		
	OR	95% CI	*P*-value	OR	95% CI	*P*-value	OR	95% CI	*P*-value
**FII (continuous**)	1.01	1.01, 1.01	<.001	1.02	1.01, 1.02	<.001	1.01	1.01, 1.02	<.001
**FII**									
Low	—	—		—	—		—	—	
Medium	1.08	1.01, 1.15	.015	1.13	1.06, 1.20	<.001	1.11	1.03, 1.20	.010
High	1.13	1.06, 1.20	<.001	1.24	1.16, 1.32	<.001	1.21	1.11, 1.32	<.001
* P* for trend			<.001			<.001			<.001

Model 1: no covariates were adjusted.

Model 2: adjusted for age, sex, and race.

Model 3: adjusted for age, sex, race, Marital status, FIR, education, CVD, excessive drinking, and smoke status.

CI = confidence interval, FII = food inflammation index, MAFLD = metabolic dysfunction-associated fatty liver disease, OR = odds ratio..

### 3.3. Subgroup analysis of the association between FII and MAFLD

**Figure 3.**
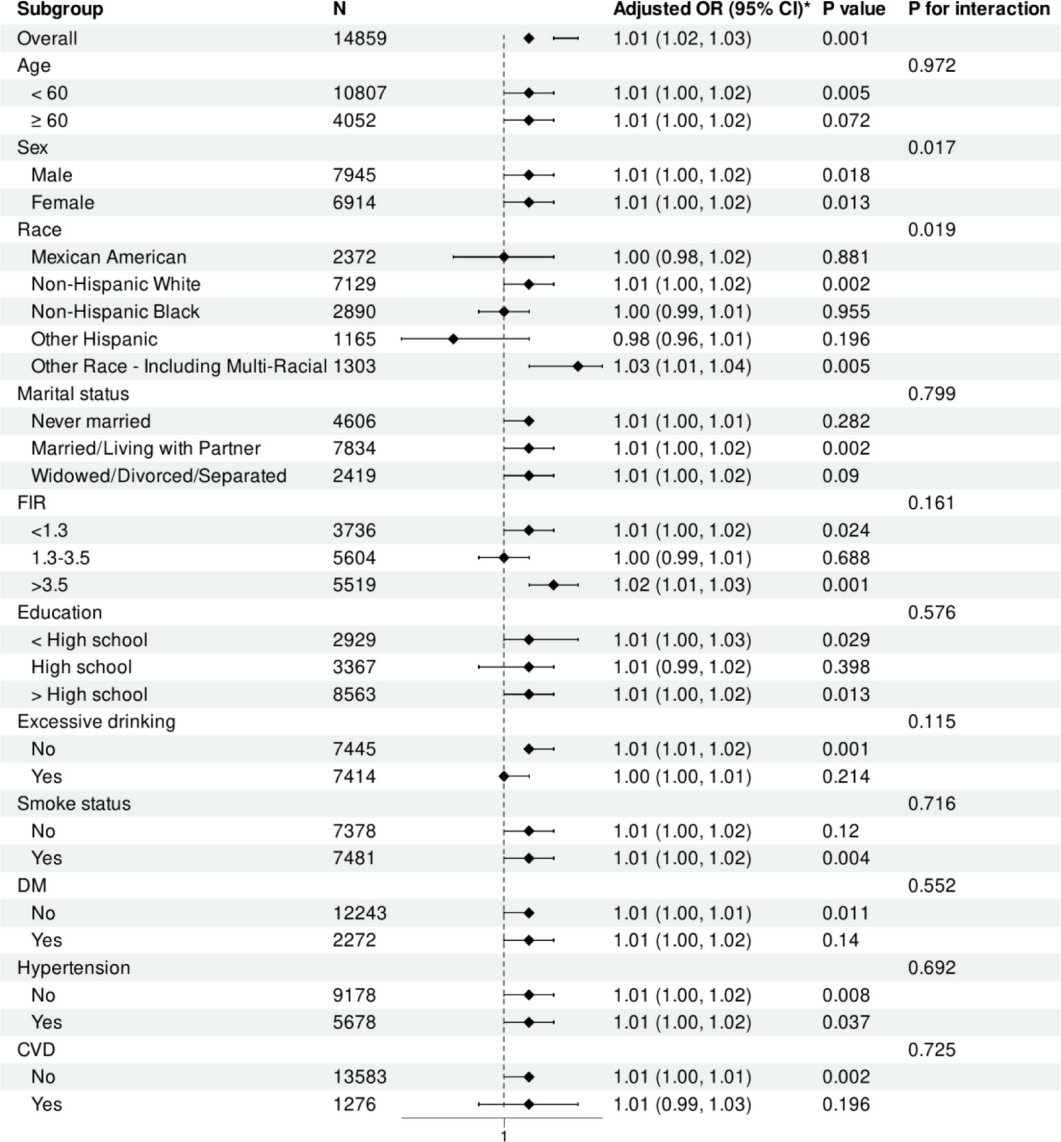
Subgroup analysis of the association between FII and MAFLD.

Subgroup analyses were conducted to assess the robustness of the association between FII and MAFLD prevalence, as illustrated in Figure 3. Interactions with age, sex, race, marital status, FIR, education, excessive drinking, smoke status, DM, hypertension, and CVD were examined to evaluate potential effect modifications. The results revealed a significant interaction in the sex subgroup (*P* for interaction = .017). Additionally, a significant interaction was noted in the race subgroup (*P* for interaction = .019), where the association was most pronounced in the “Other Race” subgroup (OR = 1.03, 95% CI:1.01–1.04, *P* = .005) and non-Hispanic White individuals (OR = 1.01, 95% CI: 1.00–1.02, *P* = .002), but not significant in Mexican Americans or non-Hispanic Black individuals. No significant interactions were observed in other subgroups, such as marital status, FIR, education, excessive drinking, smoke status, DM, hypertension, or CVD (all *P* for interaction > .05), indicating a relatively stable relationship between FII and MAFLD across these variables.

**Figure 4.**
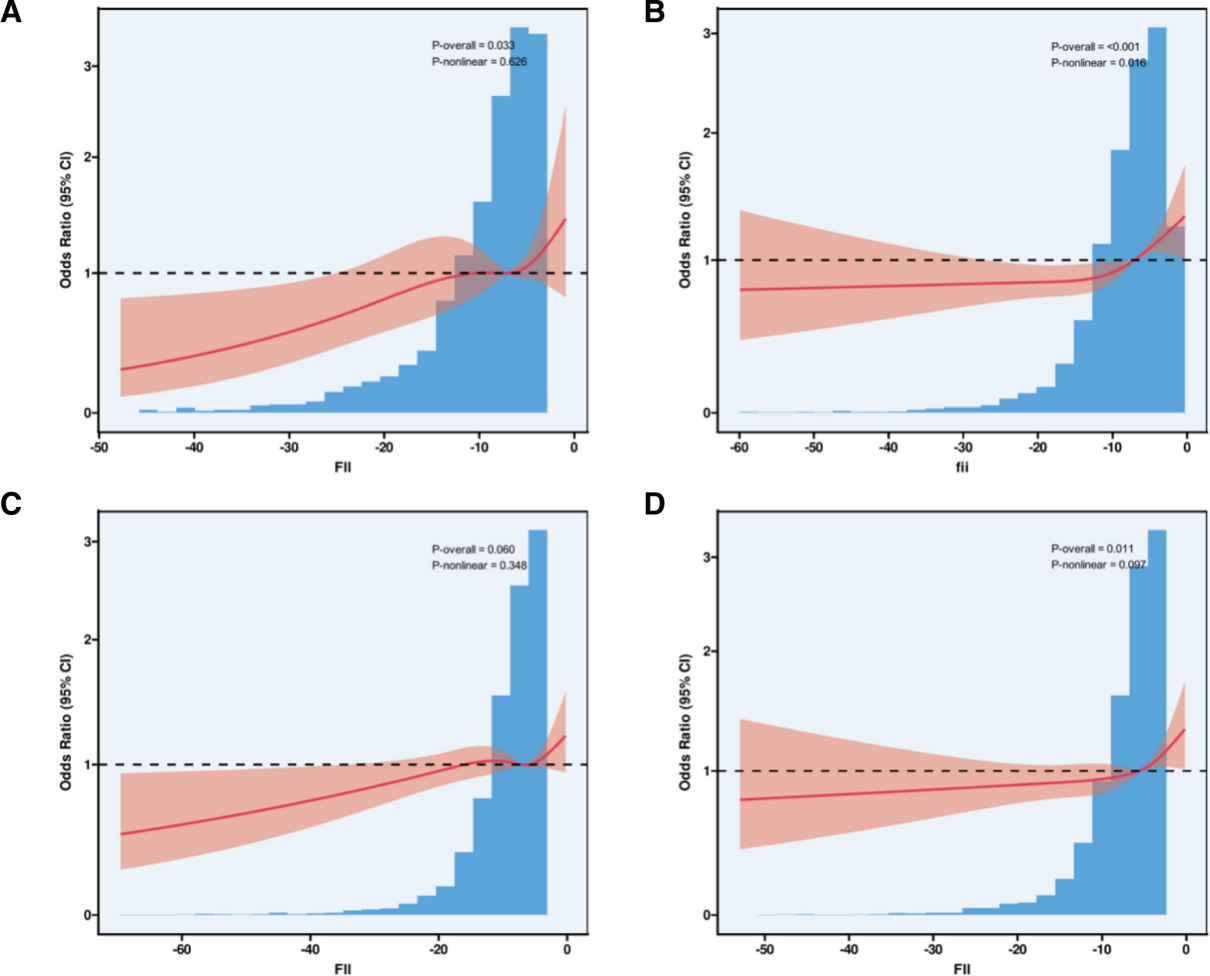
Associations between FII and MAFLD Prevalence: Restricted cubic spline (RCS) Subgroup analysis by ethnicity and sex. (A) Other race subgroup, (B) Non-Hispanic white subgroup, (C) Male subgroup (D) Female subgroup.

Furthermore, we performed RCS analyses to investigate potential nonlinear relationships between FII and MAFLD prevalence within subgroups stratified by race and sex (Fig. 4). The results revealed a significant overall association between FII and MAFLD prevalence in the ‘Other Race’ subgroup (*P*-value = .033, *P*-nonlinear = .626), the non-Hispanic White subgroup (*P*-value < .001, *P*-nonlinear = .016), and the female subgroup (*P* = .011, *P*-nonlinear = .097). Notably, a significant nonlinear association was observed in the non-Hispanic White subgroup and the female subgroup, suggesting that the relationship between FII and MAFLD may vary in a nonlinear manner within these populations. In contrast, no significant overall association was detected in the male subgroup (*P*-value = .060, *P*-nonlinear = .348), indicating that the relationship between FII and MAFLD may be less pronounced or absent in males. These findings highlight potential variations in the FII-MAFLD relationship across different demographic subgroups, with notable nonlinear trends in specific populations.

Additionally, we conducted a threshold effect analysis to examine the potential nonlinear relationship between FII and MAFLD in the non-Hispanic White subgroup. The results, presented in Table 3, indicate a significant break-point at FII = −11.560. When fitting the data using a piecewise logistic regression model, we found that for FII values < −11.560, there was no significant association with MAFLD (OR = 1.00, 95% CI: 0.99–1.01, *P*-value = .731). However, for FII values ≥ −11.560, a significant positive association was observed (OR = 1.03, 95% CI: 1.01–1.05, *P*-value = .004). The log-likelihood ratio test further supported the presence of a threshold effect (*P*-value = .042). These findings suggested that the relationship between FII and MAFLD may exhibit a threshold effect in the non-Hispanic White subgroup, with a significant association only above a certain FII level.

**Table 3:** Threshold effect analysis of FII on MAFLD.

	OR (95% CI)*	*P*-value
**Fitting by standard Logistic regression model**	1.01 (1.01, 1.02)	<.001
**Fitting by piecewise Logistic regression model (Break-Point = **−**11.560**)		
FII < −11.560	1.00 (0.99, 1.01)	.731
FII ≥ −11.560	1.03 (1.01, 1.05)	.004
**Log likelihood ratio**		**.042**

FII = food inflammation index, MAFLD = metabolic dysfunction-associated fatty liver disease.

* Adjusted for age, sex, race, Marital status, FIR, education, CVD, excessive drinking, and smoke status.

## 4. Discussion

Our study provided strong evidence of a positive association between the FII and the risk of MAFLD in a nationally representative U.S. population. Notably, a complex nonlinear relationship was observed between FII and MAFLD, with an inflection point of −11.56 identified in the non-Hispanic White subgroup. Based on these findings, we recommend reducing the consumption of processed foods such as processed meats, formulated nutrition beverages, energy drinks, sports drinks, alcoholic beverages, and nonalcoholic beverages, while increasing the intake of less processed or natural foods, including beef, fish, and dairy products.

To date, no prior studies have investigated the relationship between MAFLD and FII.. Previous studies have shown some correlation between DII and MAFLD,^[19]^ and cross-sectional studies using multiple measures of diet quality suggested that higher food quality is linked to a lower likelihood of MAFLD.^[12]^ These observations likely reflect a complex interplay of pathophysiological mechanisms through which diet-induced metabolic inflammation contributes to MAFLD development and progression. Key processes include activation of hepatic inflammatory cascades via NF-κB and NLRP3 inflammasome signaling, which may increase pro-inflammatory cytokines (TNF-α, IL-6, IL-1β), impair insulin signaling, and promote lipogenesis.^[29]^ Dietary-induced endoplasmic reticulum stress and mitochondrial dysfunction may further disrupt hepatic lipid metabolism by activating SREBP-1c and inhibiting β-oxidatio.^[30]^ Our analysis suggested a potential threshold (FII = -11.56) at which compensatory mechanisms may become insufficient to maintain metabolic homeostasis. These results indicate that FII could provide a reference point for further research on dietary interventions aimed at reducing metabolic inflammation and MAFLD risk.

Furthermore, our stratified analyses uncovered important demographic variations in the FII-MAFLD relationship. The stronger association observed in women may reflect sex-specific differences in adipokine profiles and body fat distribution, while the racial disparities could stem from genetic polymorphisms in lipid metabolism genes (e.g., PNPLA3) combined with cultural dietary patterns.^[31–34]^ These findings underscored the need for personalized nutritional approaches that account for individual characteristics in MAFLD prevention strategies.

The clinical implications of our findings are substantial. The FII could serve as a practical tool for identifying individuals at high risk for MAFLD who might benefit from targeted dietary interventions. Specifically, maintaining FII scores below the identified threshold may help prevent MAFLD development, while therapeutic reductions in FII could potentially ameliorate existing disease. These possibilities warranted investigation in randomized controlled trials of antiinflammatory dietary patterns.

This study benefited from several important strengths. First, our analysis of a large, nationally representative NHANES sample enhanced the generalizability and reliability of the findings. Second, The cross-sectional design precludes establishment of causal relationships between dietary inflammatory potential and MAFLD development - longitudinal studies with repeated dietary assessments would be valuable to confirm our observations. While 24-hour dietary recalls provide standardized data collection, they may not fully captured participants’ habitual dietary patterns due to day-to-day variability in food intake. Additionally, the NHANES database lacks comprehensive medication information, which represents an important limitation given that many MAFLD patients receive pharmacological treatments that could influence disease progression. Finally, while we adjusted for major known confounders, residual confounding by unmeasured variables remains possible. Future research should incorporate longitudinal designs, multiple dietary assessments, and more comprehensive medication data to further elucidate the relationship between dietary inflammation and MAFLD risk. Despite these limitations, our findings provided valuable insights into the potential role of diet-induced inflammation in MAFLD pathogenesis at a population level.

## 5. Conclusions

A lower FII was associated with a lower prevalence of MAFLD. The impact of FII on MAFLD prevalence was more pronounced among non-Hispanic whites compared to other racial groups. An FII score of < −11.56 for daily food intake was associated with a reduced risk of MAFLD in non-Hispanic whites. These findings advance our understanding of MAFLD pathogenesis and support the development of personalized, food-based strategies for MAFLD prevention and management. The FII framework offers a novel approach to quantifying dietary inflammatory potential that could be readily translated into clinical and public health practice.

## Acknowledgments

The authors gratefully thank the NHANES of Centers for Disease Control and Prevention for providing the publicly available data.

## Author contributions

**Data curation:** Shiyan Wang, Yu Wang.

**Formal analysis:** Shiyan Wang.

**Writing – original draft:** Shiyan Wang, Yu Wang.

**Writing – review & editing:** Hui Peng, Jin Wang, Enqiang Chen.

**Conceptualization:** Jin Wang, Enqiang Chen.

**Project administration:** Enqiang Chen.
